# Association between Variant Y402H in Age-Related Macular Degeneration (AMD) Susceptibility Gene *CFH* and Treatment Response of AMD: A Meta-Analysis

**DOI:** 10.1371/journal.pone.0042464

**Published:** 2012-08-14

**Authors:** Han Chen, Ke-Da Yu, Ge-Zhi Xu

**Affiliations:** 1 Department of Ophthalmology, Eye, Ear, Nose and Throat Hospital of Fudan University, Shanghai, People's Republic of China; 2 Shanghai Cancer Center and Cancer Institute, Fudan University, Shanghai, People's Republic of China; Duke University, United States of America

## Abstract

**Purpose:**

To investigate the association between polymorphism rs1061170 (T1277C, Y402H) in age-related macular degeneration (AMD) susceptibility gene *Complement Factor H* (*CFH*) and treatment response of neovascular AMD.

**Methods:**

We performed a literature-based meta-analysis including 10 published association studies involving 1,510 patients. Treatments included anti-VEGF (bevacizumab and ranibizumab) or photodynamic therapy. Summary odds ratios (ORs) and 95% confidence intervals (CIs) were estimated using fixed- and random-effects models. Q-statistic test was used to assess heterogeneity.

**Results:**

Polymorphism rs1061170 showed a significant summary OR of 1.68 (95% CI, 1.09 to 2.60; P = 0.020; CC versus TT; random-effects) for treatment response of neovascular AMD with heterogeneity of 0.09. In subgroup analysis, rs1061170 was more likely to be a predictor of response to anti-VEGF therapy (P = 0.011). However, heterozygous TC genotype was not associated with altered treatment response (OR = 1.18, 95% CI, 0.95 to 1.47; P = 0.145; fixed-effects). Influence analysis indicated the robustness of our findings.

**Conclusions:**

rs1061170 might be associated with treatment response of neovascular AMD, especially for the anti-VEGF agents. It might be the first meta-analytically confirmed genetic marker predictive for AMD treatment response though a further validation in larger studies is needed.

## Introduction

Age-related macular degeneration (AMD) is the leading cause of irreversible blindness among elderly population. There are two types of AMD, one is non-exudative (dry or atropic) AMD, and the other is exudative (wet or neovascular). The later form, neovascular AMD, is characterized by the presence of choroidal neovascularization (CNV) beneath the fovea. [1], [2] CNV is a pathologic ocular occurrence in which aberrant blood vessels expand from the choroid to the retinal pigment epithelium, reaching the retina in a high proportion of patients. In addition, while traditionally neovascular AMD was characterized by CNV, advances in imaging technology have also identified retinal angiomatous proliferative (RAP) lesions, which in part originate in the retina in some patients. Neovascular AMD is caused by genetic and various environmental factors such as age, smoking, and serum level of cholesterol. [1] Over the past several years, some genes have been identified to be AMD susceptibility gene. [1], [2] Of these, the *Complement Factor H* (*CFH*) gene is one of the most important genes. [3], [4], [5] Variant rs1061170 (T1277C, Y402H) in the *CFH* gene located in the heparin and CRP-binding domain may cause complement dysregulation and lead to the pathogenesis of AMD. [2], [6] Many independent studies as well as a meta-analysis have indicated that harboring the risk C-allele results in an approximately 2-fold increased risk of AMD. [3], [4], [5], [7]

The vision loss due to CNV development in neovascular AMD can be mainly managed by two different therapeutic strategies. One is photodynamic therapy (PDT) with verteporfin, and the other is intravitreal administration of drugs acting against vascular endothelial growth factor (VEGF), known as anti-VEGF treatment. Anti-VEGF agents (i.e., ranibizumab and bevacizumab) have shown efficacy in counteracting the damage owing to choroidal neovessels. Many efforts are underway to identify clinical, genetic, and pharmacologic biomarkers that could predict response to therapy, thereby providing important information to clinical decision making and treatment option. New pharmacogenetic knowledge provides data regarding the role of several single nucleotide polymorphisms (SNPs) as genetic predictors of treatment responsiveness to PDT as well as to anti-VEGF therapy. Since immune factors and inflammation are relatively important concepts linked to AMD and the complement system is a key component in the pathogenesis of AMD, [8] they may play a major role in therapeutic interventions. Previous studies proposed that patients with the variant rs1061170 in the *CFH* gene have higher background levels of inflammation, may continue to affect the disease progression, and probably lead to more rapid recurrence of neovascularization. [9] Patients with the different rs1061170 genotypes may also respond differently to treatment, and even require additional injections of agents.

Thus far, several association studies regarding the predictive role of rs1061170 in treatment response of neovascular AMD have been reported, [9], [10], [11], [12], [13], [14], [15], [16], [17], [18] though the results were inconclusive yet. Therefore, we carried out a meta-analysis focusing on the relationship between polymorphism rs1061170 and treatment response of AMD in order to get a more convincing and precise conclusion.

## Methods

### Study identification and data extraction

**Figure 1:**
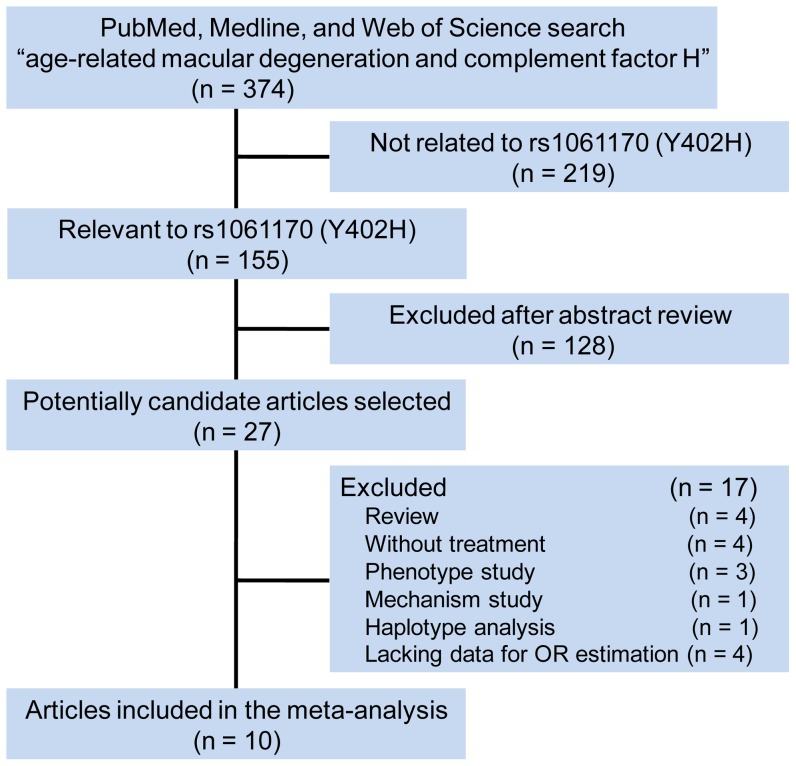
The literature search process.

Publication search of this meta-analysis was performed as described previously. [19], [20], [21] Briefly, relevant studies were searched in the PubMed, Medline, and Web of Science database (updated to February-10, 2012) using the following search terms: (“CFH” or “complement factor H”) and (age-related macular degeneration). There were 374 results, 155 of which regarding rs1061170 (T1277C, Y402H) of the *CFH* gene. Only those published studies in English language with full text articles were included in this meta-analysis; we did not define the minimum number of patients to be included for meta-analysis. The abstracts of those crudely identified 155 articles were reviewed. The inclusion criteria were: (i) evaluating the relationship between rs1061170 and treatment response of neovascular AMD, (ii) independent retrospective or prospective association study, and (iii) with sufficient available data to estimate an odds ratio (OR) with 95% confidence interval (CI). As a result, we identified 10 candidate studies for systematic review (**Figure 1**). The following variables were extracted from each study if available: first author's surname, publication year, ethnicity, numbers of cases, OR with 95% CI of response to treatment, treatment modality, and information of comparison. The information was collected independently by the two authors (C.H. and X.G.Z.), and any discrepancy were resolved by discussion.

### Statistical methods

For each study, OR with 95% CI were recorded or calculated to assess the relation strength between rs1061170 genotype and treatment response. The pooled OR was calculated by a fixed-effects model (using the Mantel-Haenszel method) or a random-effects model (using the DerSimonian and Laird method) according to the heterogeneity. Heterogeneity assumption was checked by the Q test. If P-value was not more than 0.10, the inter-study heterogeneity was considered to be significant and we would choose the random-effects model to pool the ORs. Otherwise, the fixed-effects model was employed. Because of the limitation of original data, two types of OR were calculated in present meta-analysis: TC (YH) genotype versus TT (YY) genotype, and CC (HH) genotype versus TT (YY) genotype. The potential publication bias was examined visually in a funnel plot of ln[OR] against its standard error (SE), and the degree of asymmetry was tested using Egger's test. We also performed sub-population analysis by treatment and ethnicity. Influence analysis (sensitivity analysis) was conducted by omitting each study to find potential outliers. All of the statistical analyses were performed using Stata/SE version 10.0 (Stata Corporation, College Station, TX).

## Results

### Systematic review of eligible studies

We identified 10 eligible studies for the present systematic review (**Table 1**) [9], [10], [11], [12], [13], [14], [15], [16], [17], [18]. Other four candidate studies [22], [23], [24], [25], although also investigating the relationship between neovascular AMD treatment response and rs1061170 genotype, failed to provide sufficient original data for OR estimation and were thus excluded from meta-analysis. Most reported studies were of a relative small sample size, with mean subject number of 151 (range, 86–265). We recorded the genotypic risks. If some data in the original papers were not presented in an appropriate form for meta-analysis, we estimated the ORs with 95% CIs from the raw data. Regarding ethnicity, seven studies were performed in Caucasians, one in Asians, and the remainders were unknown. The frequencies of variant C-allele of rs1061170 (T is the ancestral allele) were similar among Caucasian studies, from 50% to 58%. In contrast, the Asian study reported a much lower C-allele frequency of 7%, [16] which however was consistent with that in Asian population according to the public dbSNP and HapMap databases. All the subjects included in the meta-analysis are patients, and we need to not check the departure from Hardy-Weinberg equilibrium (HWE). [26], [27] It is because that departure from HWE in patients might indicate a genetic association between studied locus and disease rather than a biased population selection. Testing for HWE in patients is not meaningful for quality control. Of note, due to heterogeneity between studies, some studies did not directly evaluate the treatment response by comparing responders with non-responders, but used the disease progression or recurrence as a surrogate of poor response. We clarified the OR of comparison and definition of good/poor response in **Table 1**. In terms of predictive role of rs1061170 in treatment response, when TC genotype versus TT genotype, six of the ten studies showed C-allele tended to be a predictor of poor response; when CC genotype versus TT genotype, seven of nine studies indicated a resistant role of C-allele in treatment.

**Table 1:** Characteristics of the studies included in the meta-analysis.

Year	Author	Treatment	Frq*	Total	Ethnicity	OR (95% CI)		Study endpoints	OR in MTA	Definition of good response
						TC vs.TT	CC vs.TT			
2007	Brantley [10]	Bev.	0.55	86	CA	0.84 (0.17–4.09)	8.50 (0.93–105.50)	VA (Snellen)	Rspr vs. non-Rspr	Not clearly defined
2007	Seitsonen [11]	PDT	0.58	88	CA	0.66 (0.15–3.08)	0.62 (0.12–3.34)	VA (Snellen/ETDRS)	Rspr vs. non-Rspr	Not clearly defined
2008	Klein [12]	AO&Zn	0.53	239	CA	1.96 (0.71–6.24)	5.09 (1.79–16.45)	Progression	Progr. vs. no Progr.	No progression to advanced AMD after treatment
2009	Feng [13]	PDT	0.54	265	CA	1.62 (0.78–3.32)	1.77 (0.79–3.98)	VA (Snellen)	Rspr vs. non-Rspr	An improved/unchanged VA, or lost <3 lines of vision (final VA ≥6/60)
2009	Lee [9]	Ran.	0.54	156	CA	1.25 (0.94–1.67)	1.37 (1.01–1.87)	VA, recurrence	Recur. vs. no Recur.	No recurrence in follow-up time (the first 70 days excluded)
2011	Kloeckener -Gruissem [14]	Ran.	0.50	122	CA	0.68 (0.26–1.77)	2.67 (0.85–8.58)	VA (ETDRS)	Rspr vs. non-Rspr	Improve VA at ≥75^th^ percentile
2011	Nischler [15]	Bev.	0.48	197	UD	1.26 (0.53–2.95)	2.39 (0.76–8.29)	VA	Rspr vs. non-Rspr	Gained ≥3 lines in reading VA
2011	Tsuchihashi [16]	PDT	0.07	110	AA	1.41 (0.38–5.28)	NA	VA, recurrence	Recur. vs. no Recur.	No recurrence in 1-year after PDT
2012	McKibbin [17]	Ran.	0.50	104	CA	0.41 (0.13–1.19)	0.42 (0.12–1.48)	VA (ETDRS)	Rspr vs. non-Rspr	More than 5 BCVA letter score gain after 6 months
2012	Orlin [18]	Ran/Bev	0.47	143	UD	1.07 (0.45–2.60)	1.42 (0.51–3.97)	VA (Snellen)	Rspr vs. non-Rspr	An improved/unchanged VA in ≥1 eye (most recent VA ≥20/200)

Abbreviations: AA, Asian; AMD, age-related macular degeneration; AO&Zn, antioxidants and Zinc; Bev., bevacizumab; CA, Caucasian; CI, confidence interval; ETDRS, Early Treatment Diabetic Retinopathy Study; Frq, frequency; MAF, minor allele frequency; MTA., meta-analysis; NA, not available; OR, odds ratio; PDT, photodynamic therapy; Progr., progression; Ran., ranibizumab; Recur., recurrence; Rspr, responders; UD, Undeclared; VA, visual acuity.

* Frequency of the variant C-allele of rs1061170 (T1277C, Y402H). The ancestral allele of rs1061170 is T according to dbSNP (http://www.ncbi.nlm.nih.gov/projects/SNP/snp_ref.cgi?rs=1061170).

### Meta-analysis

**Figure 2:**
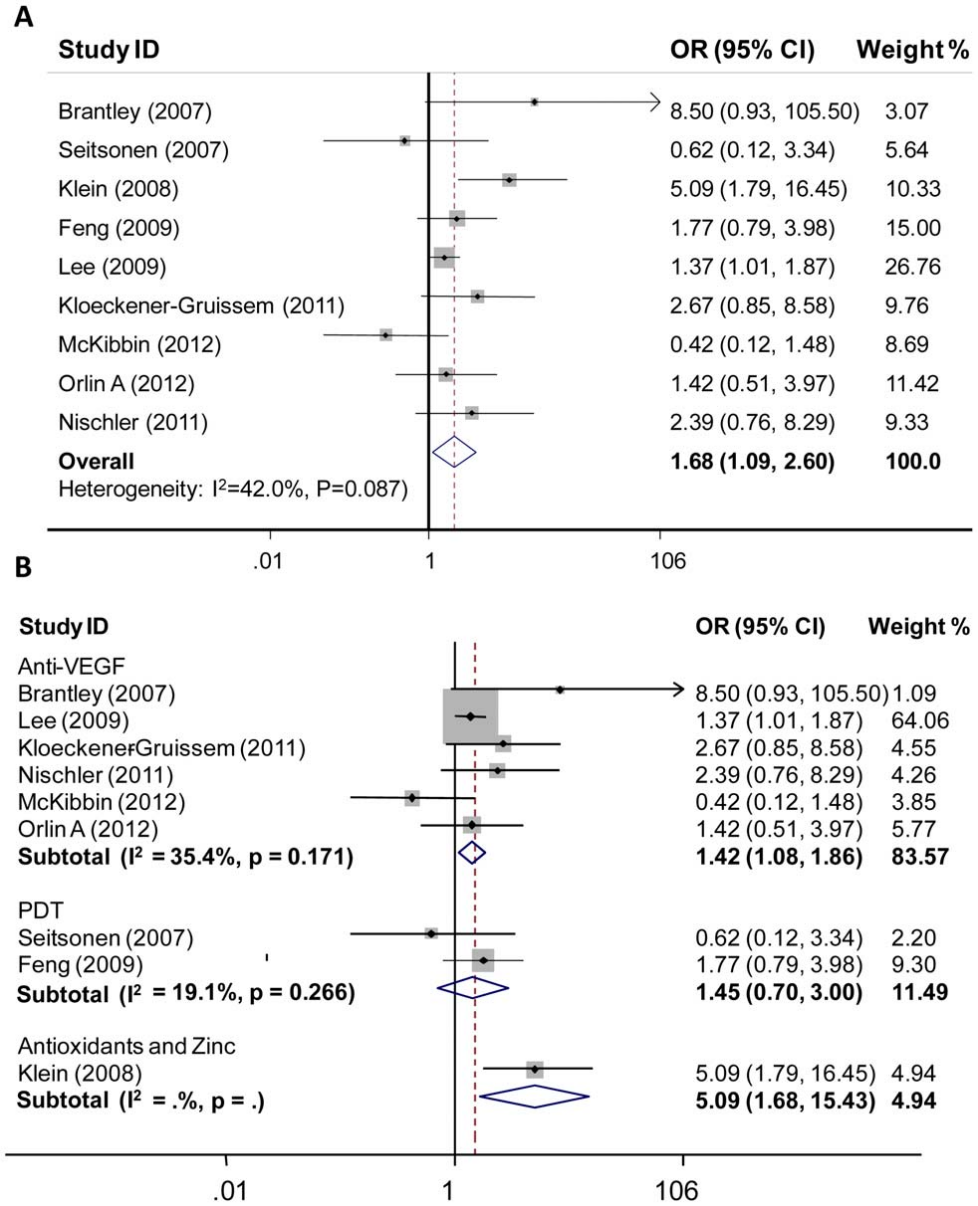
Forest plot of meta-analysis of association between *CFH* rs1061170 (T1277C, Y402H) and treatment response of neovascular AMD. **A**, Each study is shown by the point estimate of the odds ratio (OR) (the size of the square is proportional to the weight of each study) and 95% confidence interval (CI) for the OR (extending lines). Genotype comparison: CC versus TT. **B**, Forest plot of meta-analysis according to treatment modality. Anti-VEGF agents indicate bevacizumab and ranibizumab; PDT, photodynamic therapy. Genotype comparison: CC versus TT.

We subsequently meta-analyzed the ten studies for the pooled association between treatment response of neovascular AMD and rs1061170 genotype. The combined sample size was 1,510. **Table 2** presents the results of meta-analysis (ten studies for TC versus TT and nine studies for CC versus TT). The patients harboring homozygous for variant C-allele (CC genotype) seemed to be associated with a reduced response to treatment of neovascular AMD (pooled OR = 1.68, 95% CI, 1.09 to 2.60, random-effects, **Figure 2A**). However, heterozygous (TC genotype) was not associated with altered treatment response (pooled OR = 1.18, 95% CI, 0.95 to 1.47, fixed-effects). Due to limitation of original data, we could not do meta-analysis under the dominant or recessive genetic model. In subgroup analysis by treatment, we found that rs1061170 was more likely to be a predictor of anti-VEGF therapy rather than a powerful predictor for PDT effectiveness (P = 0.011; OR = 1.42, 95% CI, 1.08 to 1.86) (**Figure 2B**). When we divided the patients according to ethnicity (Caucasians or not), the association of treatment response with rs1061170 was also observed. CC genotype was associated with a reduced response to treatment of neovascular AMD with an OR of 1.73 (95% CI, 1.05 to 2.86) in Caucasians. Since only one study performed in Asians was reported, we could not conduct a sub-meta-analysis in Asians.

**Table 2:** Results of meta-analysis for the *CFH* rs1061170 (T1277C, Y402H) and treatment response of neovascular AMD.

	TC versus TT (*n* * = 10)				CC versus TT (*n* * = 9)			
	OR	95% CI	P for test	P for heterogeneity	P	95% CI	P for test	P for heterogeneity
**Overall**								
**Fixed-effects model**	1.18	0.95 to 1.47	0.145	0.597	1.52	1.19 to 1.94	0.001	0.087
**Random-effects model**	/	/	/		1.68	1.09 to 2.60	0.020	0.087
**Treatment (Fixed-effects)**								
**Anti-VEGF**	1.12	0.88 to 1.42	0.370	0.419	1.42	1.09 to 1.86	0.011	0.171
**PDT**	1.38	0.77 to 2.47	0.283	0.576	1.45	0.70 to 3.00	0.318	0.266
**Ethnicity (Random-effects)**								
**Caucasians** &	1.17	0.91 to 1.50	0.229	0.402	1.73	1.05 to 2.86	0.032	0.055

Abbreviations: CI, confidence interval; PDT, photodynamic therapy; OR, odds ratio; VEGF, vascular endothelial growth factor.

* *n* represents the number of studies included.

& including seven studies in Caucasians and one study reported by institution in Austria [15]. The study in Asians [16] is not included; one study reported by institutions in South Korea and the USA [18] is also excluded because of a concern of the mixture of Korean patients in that study.

**Figure 3:**
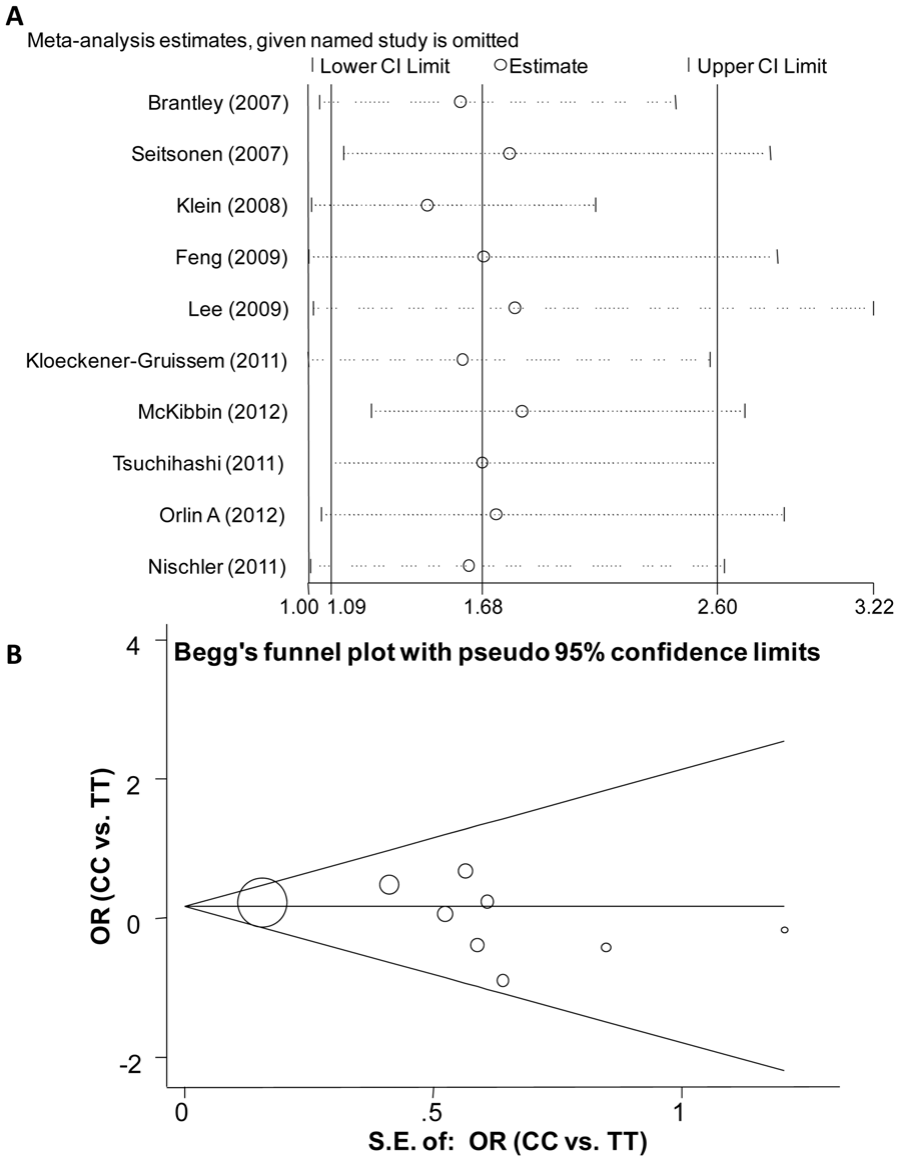
Influence analysis and publication bias plot for meta-analysis. ***A*** shows the influence of individual studies on the summary OR (CC genotype versus TT genotype). The horizontal axis represents the odds ratio. Every circle indicates the pooled OR when the left study is omitted in this meta-analysis. The two ends of every broken line represent the respective 95% CI. ***B*** shows the Begg's funnel plot of studies included in the meta-analysis (genotype comparison: CC versus TT). The vertical axis represents ln[OR] and the horizontal axis means the standard error of ln[OR]. Horizontal line and sloping lines in funnel plot represent summary OR and expected 95% CI for a given standard error, respectively. Area of each circle represents the contribution of each study to the pooled OR.

In addition, we evaluated the influence of any individual study on the overall OR for rs1061170 (**Figure 3A**). The results showed that no study fundamentally changed the positive relationship between rs1061170 and treatment response. Moreover, Begg's funnel plot and Egger's test were performed to evaluate the publication bias of literature, and no significant publication bias was observed either in the CC versus TT comparison (**Figure 3B**, P = 0.27 for Egger's test) or the TC versus TT comparison (data not shown).

## Discussion

Because relatively small sample sizes in studies have hindered reliable assessment of the association between polymorphism rs1061170 (T1277C, Y402H) in the *CFH* gene and treatment response of neovascular AMD, we performed the current systematic meta-analysis of 10 relevant studies involving 1,510 patients. The results showed that polymorphism rs1061170 was a predictor of treatment response of neovascular AMD, especially for anti-VEGF agents. Individuals harboring homozygous for the variant risk C-allele corresponded to a decreased response to treatment by approximately 1.6-fold when compared with patients carrying homozygous for the ancestral T-allele. Sensitivity analysis indicated the robustness of our findings, and evidence of publication bias was not observed either. Our study for the first time provides evidence of an association between rs1061170 and treatment response of neovascular AMD.

Complement dysregulation has emerged as an important pathogenetic factor in AMD. CFH is a critical negative regulator of complement activation. [28] Previous biological studies have demonstrated that SNP rs1061170, which is located in the coding sequence, exerts allelic differences on the binding affinity to C-reactive protein (CRP), with the risk allele showing reduced affinity. Previous observations support the functional relevance of rs1061170 to AMD pathogenesis as well as treatment response. A recent meta-analysis and many original papers have provided substantial evidence that rs1061170 is significantly associated with AMD risk. [3], [4], [5], [7] In the present study, our results solidly expand the predictive role of rs1061170 in neovascular AMD treatment response, especially for anti-VEGF therapy.

However, considering the relative low OR of 1.7 with 95% CI of 1.1 to 2.6, using rs1061170 only to predict AMD treatment response is not accurate enough, probably coupling with high false-positivity and high false-negativity. Therefore, combining a series of predictive loci as well as clinical and pathological markers would be a better way. Thus far, some polymorphisms in the *CRP* (rs2808636, rs876538), *VEGF* (rs699947, rs2146323), *pigment epithelium derived factor* (*PEDF*, rs12603825), *HTRA1* (rs11200638), and *Factor XIII-A* (rs5985) genes were also involved in prediction of neovascular AMD treatment response. Additionally, other polymorphisms (such as rs1410996, rs2274700) and haplotypes in the *CFH* gene may be also helpful to determine the treatment outcome. [16], [29] However, because of limited study number, those SNPs are not available for meta-analysis yet and further molecular epidemiological studies are needed. In addition, methodologic issues such as publication bias could have particular impact when combining results of observational studies. [30] Our results are purely based on literature that has been published and are not validated by our own data. Although we did not observe apparent publication bias by Eggers test or funnel plots, these analyses however cannot rule out this problem. Further validation in larger studies is needed.

In conclusion, our analysis might provide a piece of evidence that polymorphism rs1061170 is associated with treatment response of neovascular AMD, especially for the anti-VEGF agents (i.e., ranibizumab and bevacizumab). Our results for the first time meta-analytically confirmed a genetic marker predictive for AMD treatment response, and these results, if validated, might promote the potential for clinical treatment response prediction by combining it with other potential clinicopathological and genetic markers in the future.

## References

[1] Yachimski P, Puricelli WP, Nishioka NS (2009) Patient predictors of histopathologic response after photodynamic therapy of Barrett's esophagus with high-grade dysplasia or intramucosal carcinoma. Gastrointest Endosc 69: 205–212. 18950764 10.1016/j.gie.2008.05.032

[2] Ting AY, Lee TK, MacDonald IM (2009) Genetics of age-related macular degeneration. Curr Opin Ophthalmol 20: 369–376. 19587596 10.1097/ICU.0b013e32832f8016

[3] Haines JL, Hauser MA, Schmidt S, Scott WK, Olson LM, et al (2005) Complement factor H variant increases the risk of age-related macular degeneration. Science 308: 419–421. 15761120 10.1126/science.1110359

[4] Klein RJ, Zeiss C, Chew EY, Tsai JY, Sackler RS, et al (2005) Complement factor H polymorphism in age-related macular degeneration. Science 308: 385–389. 15761122 10.1126/science.1109557 PMC1512523

[5] Edwards AO, Ritter R 3rd, Abel KJ, Manning A, Panhuysen C, et al (2005) Complement factor H polymorphism and age-related macular degeneration. Science 308: 421–424. 15761121 10.1126/science.1110189

[6] Laine M, Jarva H, Seitsonen S, Haapasalo K, Lehtinen MJ, et al (2007) Y402H polymorphism of complement factor H affects binding affinity to C-reactive protein. J Immunol 178: 3831–3836. 17339482 10.4049/jimmunol.178.6.3831 PMC4853917

[7] Cheung CM, Tai ES, Kawasaki R, Tay WT, Lee JL, et al (2011) Prevalence of and Risk Factors for Age-Related Macular Degeneration in a Multiethnic Asian Cohort. Arch Ophthalmol 10.1001/archophthalmol.2011.376 22159171

[8] Ding X, Patel M, Chan CC (2009) Molecular pathology of age-related macular degeneration. Prog Retin Eye Res 28: 1–18. 19026761 10.1016/j.preteyeres.2008.10.001 PMC2715284

[9] Lee AY, Raya AK, Kymes SM, Shiels A, Brantley MA Jr (2009) Pharmacogenetics of complement factor H (Y402H) and treatment of exudative age-related macular degeneration with ranibizumab. Br J Ophthalmol 93: 610–613. 19091853 10.1136/bjo.2008.150995 PMC3490485

[10] Brantley MA Jr, Fang AM, King JM, Tewari A, Kymes SM, et al (2007) Association of complement factor H and LOC387715 genotypes with response of exudative age-related macular degeneration to intravitreal bevacizumab. Ophthalmology 114: 2168–2173. 18054635 10.1016/j.ophtha.2007.09.008

[11] Seitsonen SP, Jarvela IE, Meri S, Tommila PV, Ranta PH, et al (2007) The effect of complement factor H Y402H polymorphism on the outcome of photodynamic therapy in age-related macular degeneration. Eur J Ophthalmol 17: 943–949. 18050121 10.1177/112067210701700612

[12] Klein ML, Francis PJ, Rosner B, Reynolds R, Hamon SC, et al (2008) CFH and LOC387715/ARMS2 genotypes and treatment with antioxidants and zinc for age-related macular degeneration. Ophthalmology 115: 1019–1025. 18423869 10.1016/j.ophtha.2008.01.036

[13] Feng X, Xiao J, Longville B, Tan AX, Wu XN, et al (2009) Complement factor H Y402H and C-reactive protein polymorphism and photodynamic therapy response in age-related macular degeneration. Ophthalmology 116: 1908–1912 e1901. 19692124 10.1016/j.ophtha.2009.03.011

[14] Kloeckener-Gruissem B, Barthelmes D, Labs S, Schindler C, Kurz-Levin M, et al (2011) Genetic association with response to intravitreal ranibizumab in patients with neovascular AMD. Invest Ophthalmol Vis Sci 52: 4694–4702. 21282580 10.1167/iovs.10-6080

[15] Nischler C, Oberkofler H, Ortner C, Paikl D, Riha W, et al (2011) Complement factor H Y402H gene polymorphism and response to intravitreal bevacizumab in exudative age-related macular degeneration. Acta Ophthalmol 89: e344–349. 21232084 10.1111/j.1755-3768.2010.02080.x

[16] Tsuchihashi T, Mori K, Horie-Inoue K, Gehlbach PL, Kabasawa S, et al (2011) Complement factor H and high-temperature requirement A-1 genotypes and treatment response of age-related macular degeneration. Ophthalmology 118: 93–100. 20678803 10.1016/j.ophtha.2010.04.007

[17] McKibbin M, Ali M, Bansal S, Baxter PD, West K, et al (2012) CFH, VEGF and HTRA1 promoter genotype may influence the response to intravitreal ranibizumab therapy for neovascular age-related macular degeneration. Br J Ophthalmol 96: 208–212. 21558292 10.1136/bjo.2010.193680

[18] Orlin A, Hadley D, Chang W, Ho AC, Brown G, et al (2012) Association between high-risk disease loci and response to anti-vascular endothelial growth factor treatment for wet age-related macular degeneration. Retina 32: 4–9. 21878851 10.1097/IAE.0b013e31822a2c7c PMC7440363

[19] Rudnicka AR, Jarrar Z, Wormald R, Cook DG, Fletcher A, et al (2012) Age and gender variations in age-related macular degeneration prevalence in populations of European ancestry: a meta-analysis. Ophthalmology 119: 571–580. 22176800 10.1016/j.ophtha.2011.09.027

[20] Thakkinstian A, McKay GJ, McEvoy M, Chakravarthy U, Chakrabarti S, et al (2011) Systematic review and meta-analysis of the association between complement component 3 and age-related macular degeneration: a HuGE review and meta-analysis. Am J Epidemiol 173: 1365–1379. 21576320 10.1093/aje/kwr025

[21] Zhou P, Fan L, Yu KD, Zhao MW, Li XX (2011) Toll-like receptor 3 C1234T may protect against geographic atrophy through decreased dsRNA binding capacity. FASEB J 25: 3489–3495. 21712495 10.1096/fj.11-189258

[22] Goverdhan SV, Hannan S, Newsom RB, Luff AJ, Griffiths H, et al (2008) An analysis of the CFH Y402H genotype in AMD patients and controls from the UK, and response to PDT treatment. Eye (Lond) 22: 849–854. 17464302 10.1038/sj.eye.6702830 PMC5989925

[23] Brantley MA Jr, Edelstein SL, King JM, Plotzke MR, Apte RS, et al (2009) Association of complement factor H and LOC387715 genotypes with response of exudative age-related macular degeneration to photodynamic therapy. Eye (Lond) 23: 626–631. 18292785 10.1038/eye.2008.28 PMC5531276

[24] Imai D, Mori K, Horie-Inoue K, Gehlbach PL, Awata T, et al (2010) CFH, VEGF, and PEDF genotypes and the response to intravitreous injection of bevacizumab for the treatment of age-related macular degeneration. J Ocul Biol Dis Infor 3: 53–59. 21811649 10.1007/s12177-010-9055-1 PMC3148139

[25] Teper SJ, Nowinska A, Pilat J, Palucha A, Wylegala E (2010) Involvement of genetic factors in the response to a variable-dosing ranibizumab treatment regimen for age-related macular degeneration. Mol Vis 16: 2598–2604. 21151600 PMC3000236

[26] Yu KD, Shao ZM (2011) Inspection of a deviation from Hardy-Weinberg equilibrium in familial breast cancer cases from a case-control study in a meta-analysis. Breast Cancer Res Treat 127: 577–579. 21279435 10.1007/s10549-011-1361-1

[27] Ziegler A, Van Steen K, Wellek S (2011) Investigating Hardy-Weinberg equilibrium in case-control or cohort studies or meta-analysis. Breast Cancer Res Treat 128: 197–201. 21184275 10.1007/s10549-010-1295-z

[28] Skerka C, Lauer N, Weinberger AA, Keilhauer CN, Suhnel J, et al (2007) Defective complement control of factor H (Y402H) and FHL-1 in age-related macular degeneration. Mol Immunol 44: 3398–3406. 17399790 10.1016/j.molimm.2007.02.012

[29] Mori K, Horie-Inoue K, Gehlbach PL, Takita H, Kabasawa S, et al (2010) Phenotype and genotype characteristics of age-related macular degeneration in a Japanese population. Ophthalmology 117: 928–938. 20132989 10.1016/j.ophtha.2009.10.001

[30] Stroup DF, Berlin JA, Morton SC, Olkin I, Williamson GD, et al (2000) Meta-analysis of observational studies in epidemiology: a proposal for reporting. Meta-analysis Of Observational Studies in Epidemiology (MOOSE) group. JAMA 283: 2008–2012. 10789670 10.1001/jama.283.15.2008

